# Concomitant spine trauma in patients with traumatic brain injury: Patient characteristics and outcomes

**DOI:** 10.3389/fneur.2022.861688

**Published:** 2022-08-18

**Authors:** Lennart Riemann, Obada T. Alhalabi, Andreas W. Unterberg, Alexander Younsi, The CENTER-TBI investigators and participants, Cecilia Åkerlund

**Affiliations:** Department of Neurosurgery, Heidelberg University Hospital, Heidelberg, Germany

**Keywords:** traumatic brain injury, traumatic spine injury, outcome, CENTER-TBI, spine trauma

## Abstract

**Objective:**

Spine injury is highly prevalent in patients with poly-trauma, but data on the co-occurrence of spine trauma in patients with traumatic brain injury (TBI) are scarce. In this study, we used the Collaborative European NeuroTrauma Effectiveness Research in Traumatic Brain Injury (CENTER-TBI) database to assess the prevalence, characteristics, and outcomes of patients with TBI and a concurrent traumatic spinal injury (TSI).

**Methods:**

Data from the European multi-center CENTER-TBI study were analyzed. Adult patients with TBI (≥18 years) presenting with a concomitant, isolated TSI of at least serious severity (Abbreviated Injury Scale; AIS ≥3) were included. For outcome analysis, comparison groups of TBI patients with TSI and systemic injuries (non-isolated TSI) and without TSI were created using propensity score matching. Rates of mortality, unfavorable outcomes (Glasgow Outcome Scale Extended; GOSe < 5), and full recovery (GOSe 7–8) of all patients and separately for patients with only mild TBI (mTBI) were compared between groups at 6-month follow-up.

**Results:**

A total of 164 (4%) of the 4,254 CENTER-TBI core study patients suffered from a concomitant isolated TSI. The median age was 53 [interquartile range (IQR): 37–66] years and 71% of patients were men. mTBI was documented in 62% of cases, followed by severe TBI (26%), and spine injuries were mostly cervical (63%) or thoracic (31%). Surgical spine stabilization was performed in 19% of cases and 57% of patients were admitted to the ICU. Mortality at 6 months was 11% and only 36% of patients regained full recovery. There were no significant differences in the 6-month rates of mortality, unfavorable outcomes, or full recovery between TBI patients with and without concomitant isolated TSI. However, concomitant non-isolated TSI was associated with an unfavorable outcome and a higher mortality. In patients with mTBI, a negative association with full recovery could be observed for both concomitant isolated and non-isolated TSI.

**Conclusion:**

Rates of mortality, unfavorable outcomes, and full recovery in TBI patients with and without concomitant, isolated TSIs were comparable after 6 months. However, in patients with mTBI, concomitant TSI was a negative predictor for a full recovery. These findings might indicate that patients with moderate to severe TBI do not necessarily exhibit worse outcomes when having a concomitant TSI, whereas patients with mTBI might be more affected.

## Introduction

Traumatic brain injury (TBI) contributes to the global burden of disease in a sizeable manner (1). The incidence of TBI has risen in the past years (2) and is estimated to become even more relevant with increasing events of traffic accidents and falls of the elderly (3, 4).

Traumatic brain injury can be complicated by additional injuries, such as traumatic spinal injuries (TSIs). When studying patients with spinal cord injury, the rate of concomitant TBI was estimated between 40 and 74% (5, 6). TBI in most of these patients was classified as mild (7). It is postulated that in the context of spine trauma, simultaneous TBI events are underdiagnosed (8). Unsurprisingly, TBI pertaining to spinal cord injury was found to be most frequent when the cervical and thoracic spine are affected (9).

Although various reports on TBI from a spinal injury perspective exist, little is known about the converse case of concomitant isolated spine trauma in patients suffering primarily from TBI. A recent meta-analysis found the rate of concomitant TSI in patients with TBI to be at around 13%, with cervical spinal injury amounting to almost half of the injuries diagnosed (10). This consolidates previous reports on cervical spine injury in larger patient cohorts with TBI (11). Indeed, patients with severe TBI were found to be at a particularly higher risk for sustaining injuries to the cervical spine (12).

Previous literature, while epidemiologically describing the prevalence of and risk factors for concomitant TBI and TSI, rarely elucidates the neurological outcomes of affected patients. In a retrospective analysis, patients with simultaneous TBI and TSI were reported to show increased motor deficits and limited functional gains in rehabilitation (13). Nevertheless, the question whether patients with concomitant TBI and TSI bear an inherent risk for a worse neurological outcome or a higher rate of mortality has yet to be tackled by prospectively collected observational data.

This study hence aimed at assessing the prevalence and characteristics of patients with TBI and concurrent, isolated TSI and comparing outcomes of such patients with TBI only in the Collaborative European NeuroTrauma Effectiveness Research in Traumatic Brain Injury (CENTER-TBI) cohort.

## Methods

### Study design

In the present study, data collected as part of the CENTER-TBI core study were analyzed. CENTER-TBI is a European multi-center, observational, longitudinal cohort study of patients presenting with TBI of all severities. Patients were eligible for enrollment when presenting with a clinical diagnosis of TBI to a participating study center within 24 h and when a computed tomography (CT) scan was performed at admission. Informed consent was required from all patients and had to be obtained prior to enrollment. The study protocol adhered to all national and local ethical committee requirements of participating study centers. Patients were enrolled from December 2014 to December 2017 in 59 centers across Europe and Israel. More details on the CENTER-TBI study and main descriptive findings have been published elsewhere (14, 15).

### Study cohort and outcome parameters

For this study, we included adult CENTER-TBI core study patients (i.e., 18 years or older) with TBI that presented with a concomitant, isolated TSI. TSI was defined by an Abbreviated Injury Scale (AIS) score of ≥3 (indicating an injury of at least serious severity) in the cervical, thoracic, or lumbar spine. To study the impact of the TSI separately from poly-traumatic injuries, patients were excluded when also suffering from serious injuries (also defined as an AIS score of ≥3) in other body regions, namely, injuries to the thorax and chest, abdomen, pelvis, upper and lower extremities, or skin. As a complementary investigation, the same analyses were repeated for patients with non-isolated TSI, i.e., those with spine injuries (AIS scores ≥3) and concomitant injuries (AIS scores ≥3) in any of the other body regions. Primary outcome parameters were mortality [i.e., Glasgow Outcome Scale Extended (GOSe) = 1], unfavorable outcomes (i.e., GOSe < 5), and full recovery (i.e., GOSe = 7–8). All data were retrieved from the CENTER-TBI core study database in version 3.0 *via* the accessing tool Neurobot (RRID: SCR_ 017004).

### Statistical analysis

Patient characteristics were analyzed using descriptive statistics. Continuous variables are reported as medians and interquartile ranges (INRs), while ordinal and categorical variables are presented as numbers and frequencies unless stated otherwise. The completeness of data is reported in Supplementary Table S1. Prior to outcome analysis, multiple imputation with 100 imputed datasets was used to address missing data in the control variables (age, sex, baseline Glasgow Coma Scale [GCS], performed cranial surgery, intracranial CT abnormality (mass lesion, extra-axial hematoma, epidural hematoma, acute subdural hematoma, chronic and subacute subdural hematoma, a subdural collection of mixed density, contusion, traumatic axonal injury, traumatic subarachnoid hemorrhage, intraventricular hemorrhage, midline shift, or cisternal compression), and American Society of Anesthesiologists [ASA] class) and the primary outcome variables (GOSe). Missing data were assumed to be missing at random. GCS and GOSe were defined as ordinal variables. The mortality, unfavorable outcomes, and full recovery of the variables were subsequently derived from imputed GOSe scores. After multiple imputation, propensity score matching with the above-named control variables and GOSe at 6-month follow-up as outcome variable was performed to create a matched comparison group of patients with TBI without concomitant TSI. The control variables were chosen *a priori* based on clinical expertise. Matching was performed within each imputed dataset. Effect estimates of concomitant TSI to outcomes were analyzed using weighted logistic regression models in each dataset. Additionally, logistic multivariable regression with (isolated or non-isolated) TSI as predictor and adjustment for the same control variables used in the propensity score analysis were performed for the three outcomes as a complementary analysis. Finally, effect estimates from each model were pooled according to Rubin's rules (16). The statistical software R was used for all analyses (https://www.r-project.org/ - version 4.1.1) (17).

## Results

### Patient characteristics, injury details, and prehospital course

**Figure 1:**
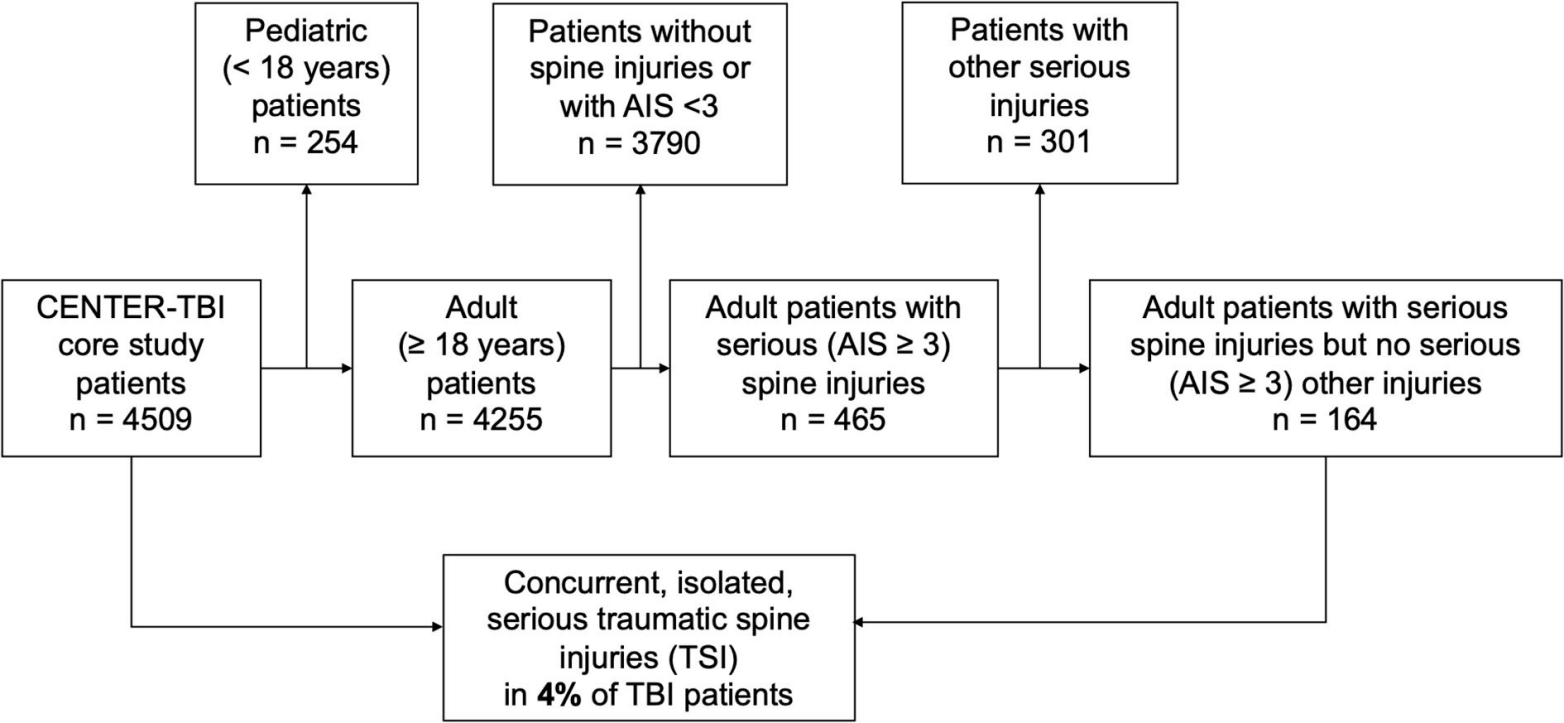
Study design. Adult patients in the Collaborative European NeuroTrauma Effectiveness Research in Traumatic Brain Injury (CENTER-TBI) database were screened. Patients with poly-trauma were excluded. Only patients sustaining TBI along with isolated traumatic spine injury (TSI) without the presence of further trauma were included in the analyses.

**Figure 2:**
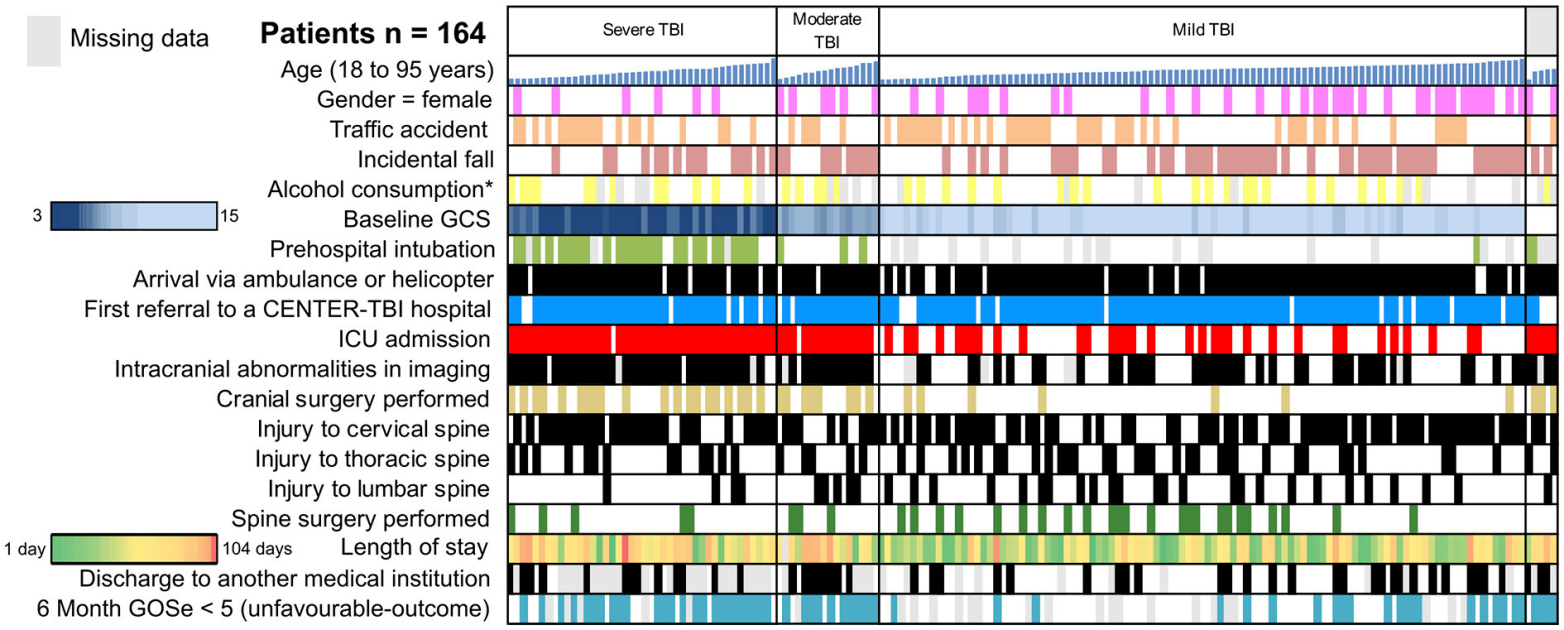
Traumatic brain injury (TBI)-traumatic spine injury (TSI) study cohort. Selected variables of the 164 patients included in the analysis are depicted. Rows represent representative variables, and each column represents one patient. Sub-cohorts are separated based on TBI severity (severe TBI: GCS < 8; moderate TBI: GCS 9–12; and mild TBI: GCS 13–15). In the sub-cohorts, patients are sorted by age from youngest (19 years) to oldest (95 years). A heat-map was utilized to visualize the length of stay (green: short, red: long) and GCS (dark blue: 15, light blue: 3). ICU, intensive care unit; GOSe, Glasgow Outcome Scale Extended. *Confirmed or suspected alcohol consumption. Gray represents missing data.

A total of 164 adult patients with TBI and concomitant TSI were included in this study, representing about 4% of the entire CENTER-TBI study population (Figure 1). The median age in this subgroup of patients with simultaneous head and isolated spine injury was 53 years (IQR: 37–66 years) and 116 (71%) were men. The majority of injuries were caused by either incidental falls (47%, *n* = 77) or by road-traffic incidents (42%, *n* = 68). Alcohol intoxication confirmed by increased alcohol blood levels was found in 16% of patients (*n* = 26) and suspected in another 8% (*n* = 13). Most patients were brought to the hospital by ambulance (76%, *n* = 123) or by helicopter (12%, *n* = 19). Some patients even presented as walk-ins or drop-offs (6%, *n* = 9). Endotracheal intubation at the scene of an accident was performed in 22% of patients (*n* = 33). In total, 86% of patients (*n* = 141) were directly transported to the study center, while the remainder were referred to the study center from another hospital (see also Figure 2).

### Clinical presentation and clinical course of TBI patients with concomitant TSI

Upon admission at the study center, severe TBI (GCS 3–8) was present in 26% of patients (*n* = 42), while moderate or mild TBI (mTBI) was documented in 10% (*n* = 16) and 62% (*n* = 101) of cases, respectively. A traumatic spine injury of at least serious severity was located in the lumbar spine in 32 patients (20%), in the thoracic spine in 51 patients (31%), and in the cervical spine in 104 patients (63%). In 21 patients (13%), more than one region of the spine was affected (e.g., both cervical and thoracic spine injuries). The majority of patients (57%, *n* = 93) were admitted to the ICU, while 63 patients (38%) were admitted to the regular ward. Among patients admitted to the ICU, the requirement for mechanical ventilation was named as the primary reason in 40 patients (43%), followed by the need for frequent neurological observations in 22 patients (24%) and neurosurgical intervention in 13 patients (14%). Spine stabilization surgery was performed in 32 patients (20%). During the hospital stay, respiratory complications were documented in 14 patients (12%), making it the most common type of complication. Further complications included seizures in 5 patients (4%), cardiac complications in 4 patients (3%), and urinary tract infections in 6 patients (5%). Patients with TBI and concomitant TSI stayed in the hospital for a median of 9 (3–20) days. Most patients could be discharged home (56%, *n* = 70), while 26 patients (21%) were discharged to a rehabilitation facility, and 24 patients (19%) were transferred to another hospital (see also Figure 2).

### Outcomes of TBI patients with concomitant TSI

In this cohort of patients with TBI and concomitant TSI, 18 of 164 patients were dead after 6 months, yielding a mortality rate of 11%. Of those, 13 died in the ICU. In 9 patients, the initial head injury was documented as the cause of death whereas secondary intracranial damage was documented in 2 patients. For the remaining deceased patients, no cause of death was documented. A total of 48 patients (29%) were considered to have an unfavorable outcome (GOSe < 5). Approximately, one-third of the patients achieved a full recovery (GOSe 7 or 8). To compare the outcomes of TBI patients with and without isolated, concomitant TSI, we performed propensity score matching with subsequent weighted logistic regression to estimate the effect of the simultaneous spine injury on patient outcomes. Patients were matched with age, sex, baseline GCS, performed cranial surgery, intracranial CT abnormalities, and ASA class as covariables (see Supplementary Tables S2, S3 for balance statistics and exemplary descriptions of the matched cohorts). In the outcome analysis, the presence of an isolated, concomitant TSI was neither significantly associated with mortality [β = −0.12 (−0.84 to 0.59), *p* = 0.732] nor with unfavorable outcomes [β = 0.28 (−0.21 to 0.77), *p* = 0.270], or full recovery [β = −0.29 (−0.76 to 0.18), *p* = 0.228]. Similar results were obtained in the logistic regression analysis (Supplementary Table S4), which showed that an isolated TSI, when controlling for age, sex, baseline GCS, performed cranial surgery, intracranial CT abnormalities, and ASA class, was neither a significant predictor of full recovery (*p* = 0.084) nor of unfavorable outcomes (*p* = 0.184) or death (*p* = 0.355) in our cohort. To put these results into a broader context, we performed a similar analysis but examined patients with TBI and concomitant TSI in conjunction with systemic injuries (i.e., non-isolated TSI) instead of an isolated TSI. In comparison with a matched cohort, TSI with concomitant systemic injuries in patients with TBI was negatively associated with full recovery (*p* ≤ 0.001), but not with unfavorable outcomes (*p* = 0.130) or mortality (*p* = 0.282). In logistic regression analysis, a TSI with systemic injuries was a significant negative predictor of full recovery (*p* < 0.001) and unfavorable outcomes (*p* = 0.003), but not mortality (*p* = 0.355; Supplementary Table S4).

When only patients with mTBI were included in a subgroup analysis, no significant associations between isolated TSI and full recovery [β = −0.468 (−1.127 to 0.190), *p*-value = 0.160] and unfavorable outcomes [β = 0.899 (−0.175 to 1.973), *p*-value = 0.099] were seen when using propensity score matching. In the logistic regression analysis, isolated TSI was a significant negative predictor of full recovery [β = −0.507 (−0.994 to 0.012), *p* = 0.042] and a predictor of unfavorable outcomes [β = 0.770 (0.145–1.394), *p* = 0.016]. In mTBI patients with TSI and systemic injuries, TSI was significantly associated with unfavorable outcomes [β = 0.853 (0.006–1.699), *p*-value = 0.048] and inversely with full recovery [β = −1.311 (−1.925 to −0.698), *p*-value < 0.001] in the propensity score-matching analysis. Similarly, in the logistic regression analysis, a TSI in mTBI patients with systemic injuries was significantly associated with unfavorable outcomes [β = 1.150 (0.610–1.691), *p* < 0.001] and, in a negative direction, with full recovery [β = −1.345 (−1.772 to −0.918), *p* < 0.001]. The outcome analysis was not performed for mortality in the subgroup analysis of patients with mTBI due to the very low mortality rate (i.e., zero, and three patients among mTBI patients with isolated and non-isolated TSI were dead at the follow-up timepoint after 6 months, respectively) in this subgroup.

## Discussion

While there is a wealth of epidemiological data on TBI studied from an SCI perspective, the potential role of a simultaneous TSI in exacerbating neurological deficits in patients with TBI remains largely unexplored. This study reported on concomitant TSI using data from a large prospectively followed up cohort of patients presenting with TBI as their main diagnosis and provided propensity-matching analyses to determine the influence of such injury on their global functional outcomes.

In this cohort, the rate of patients with TBI sustaining further isolated injury of at least serious intensity to the spine was found to be 4%. The rift between our current findings and previous analyses indicating higher rates of TSI in patients with TBI of up to 13% (10) could well be attributed to differences in the applied methodology, especially as to what is defined as an “injury.” One key difference could be the AIS used in this study. The AIS is a standardized tool to reliably classify injuries and assess their severity (18, 19). Patients with TBI were regarded to have suffered a concomitant TSI when the AIS score of the cervical, thoracic, or lumbar spine satisfied at least serious severity. In this functional outcome-oriented analysis, thresholds for defining TSI were set as a trade-off between including patients with very minor and clinically negligible injuries that would otherwise skew the analysis and over-estimate TSI in patients with TBI vs. solely including patients undergoing surgical spinal stabilization and hence overlooking patients sustaining TSI with a “relevant” burden of disease that was managed non-surgically.

In a similar vein, an analysis excluding patients showing further injuries beyond TSI was envisaged to help eliminate possible confounders through further injuries (for example, to the skeletal system), and, therefore, yield a less-biased analysis that could compare characteristics and outcomes of isolated TSI+TBI vs. TBI-only patients. Indeed, further propensity-matching and logistic regression analyses compared patients with TBI, and systemic injuries (that included TSI) did show systemic injuries to be associated with unfavorable outcomes and to prevent full recovery.

Confirming data in previous studies, more than 60% of the spinal injuries diagnosed in our cohort were cervical (10). This was previously linked to the physiological bio-mechanical proximity of the cervical spine to the head (20), rendering concomitant injury to the cervical spine in TBI cases more likely than to other regions of the spine (10). Regarding injury causes, incidental falls and road traffic accidents accounted for the majority of TBIs (47 and 40%, respectively). On the one hand, the rate of road traffic accidents seems to be higher in this cohort than what has been previously reported in (isolated) TBI in high-income countries (21), which lends grounds for speculation that road accidents (which are usually poly-traumas in nature), might contribute to an increased risk of concomitant injury, especially with previous studies showing a high proportion of SCI patients with TBI to be victims of road traffic accidents (9). In addition, motor traffic accidents and herein old age, in particular, have been associated with higher odds of cervical spine injury (22). On the other hand, the larger proportion of incidental falls confirms a worldwide trend of increasing TBI rates secondary to falls of the elderly (4). In this cohort, patients with suspected or confirmed alcohol use amounted to 26%. Indeed, alcohol has been previously shown to be the strongest risk factor for clinical TBI in patients with SCI (7), hinting at the possibility that this could be another factor that fosters concomitant injury.

Missed diagnosis of simultaneous spinal injury in patients with TBI was deemed detrimental in the past and accounted for further neurological deterioration (11), especially because patients suffering severe TBI are difficult to assess clinically and possess a higher risk of sustaining injuries to the cervical spine (12). In terms of prognosis, this observation is, however, not reflected by the data we present, in which outcomes were comparable between TBI+TSI and TBI-only patients. Rather, it is conceivable that the probability of missing relevant spine trauma in the wake of comprehensive CT and MRI imaging [that was less available 20 years earlier (11)] in the participating study centers should be low. This is further supported by the fact that in this cohort, 86% of the patients were primarily transported to a more specialized trauma center (part of the CENTER-TBI study group) where the availability of the necessary infrastructure for diagnosis and treatment of spine trauma (especially spine stabilization surgery) is expected to be higher. It is therefore advisable that given the relevant rate of TSI in patients with TBI and the complex spine surgery these patients might potentially require, patients with TBI are primarily presented to specialized trauma centers of maximum care, especially when concomitant TSI is suspected. This effect could indeed be of even more relevance in the context of patients with mTBI since our analysis demonstrated how in the case of the subgroup of patients with mTBI, isolated TSI (or TSI in conjunction with systemic injuries) does indeed hinder full recovery and negatively influence outcomes.

Although most of the patients in the TBI+TSI cohort were admitted to the ICU, there was no significant difference in mortality when comparing them to patients with isolated TBI in our study. This hints at the possibility that in TBI patients with concomitant TSI, the intracranial injury still represents the main prognosis-limiting factor, especially in severe and moderate TBI. The disparity between the findings on patients with mTBI and all patients of the cohort emphasizes on how the prognosis of patients with moderate and severe TBI is limited by their cranial injury and how TSI becomes more relevant in patients sustaining mTBI, that are otherwise less limited in terms of their neurological outcomes. The question is to whether the necessity of intubation and mechanical ventilation is a result of loss of consciousness owing to TBI or of respiratory failure secondary to injury of the cervical spine remains and cannot be explored using the data provided, although previous reports have indicated the presence of the latter patient group (12).

Similarly, two-thirds of the patients in the TBI+TSI cohort showed a favorable outcome (divided in half between complete recovery and incomplete recovery with a GOSe > 5), leaving a third with unfavorable outcomes in our current analysis. Interestingly, an older study estimated patients with the recovery of neurological function after severe and moderate TBI and concomitant cervical TSI (no mTBIs included) to be at about a third (11), which is very comparable to the data we present. Apart from that, little data have been provided in previous epidemiological studies on the specific functional outcomes of TBI patients with concomitant TSI. This once again emphasizes the importance of the data presented in this study, in which the rates of mortality and unfavorable outcomes in TBI patients with concomitant TSI were comparable to the respective rates observed in a matched group of TBI patients without TSI.

In summary, this analysis of prospective observational data sheds light on the current prognosis of patients suffering from TBI with a concomitant isolated or non-isolated TSI in the CENTER-TBI participating centers, showing an outcome that is comparable with what is known in the literature (15). The data presented underscore the role of specialized trauma care centers in preventing further neurological deterioration owing to concomitant TSI especially in patients with mTBI through early detection and adequate therapy of spine trauma.

## Limitations

Several important limitations must be noted. As an observational study focused on TBI, in general, no additional information on the exact nature of the spine injury or its treatment (that included surgical details) was recorded in the CENTER-TBI database. Thus, injuries to the spinal cord could have been present in some patients but not in others, potentially leading to a considerable heterogeneity for the variable “spine trauma.” This should be considered when interpreting our current results. Additional studies are needed to assess how different types of spine and spinal cord injuries relate to outcomes in patients with TBI. To the same end, detailed parameters assessing specifically the spine function, such as motor and sensory function of the extremities, as well as the function of the autonomic nerve system, were not available but would be desirable both for the description of the baseline clinical status and for the evaluation of recovery at follow-up. In terms of outcome analysis, the matching process is dependent on the chosen covariables and unmeasured covariables that might play an important role are not accounted for. Finally, as only TBI patients with concomitant spine trauma were included, the sample sizes of the different subgroups of patients in our analyses were limited. Larger cohorts are needed for a more robust generalizability and to possibly detect more subtle effects of a concomitant spine injury on outcomes in patients with TBI.

## Data availability statement

The data analyzed in this study was obtained from the CENTER-TBI database; the following licenses/restrictions apply: Upon a reasonable request, access to the dataset must first be reviewed and approved by the CENTER-TBI Management Committee and should be directed to https://www.center-tbi.eu/data.

## Ethics statement

Ethical approval was obtained for each recruiting site. A complete list is given on https://www.center-tbi.eu/project/ethical-approval or available as a Supplementary file. All patients had to give their written informed consent before enrollment in CENTER-TBI.

## Author contributions

Study concept and design and data collection and analysis: LR and AY. Data interpretation and reviewing and editing: LR, OA, AU, and AY. Writing the manuscript: LR and OA. Supervision: AY. All authors approved the final version of the submitted manuscript.

## Funding

CENTER-TBI was supported by the European Union 7th Framework program (EC Grant 602150). Additional funding was obtained from the Hannelore Kohl Stiftung (Germany), from OneMind (USA), and Integra LifeSciences Corporation (USA). Those funders were not involved in the study design, collection, analysis, interpretation of data, the writing of this article, or the decision to submit it for publication.

## CENTER-TBI investigators and participants

Cecilia Åkerlund^1^, Krisztina Amrein^2^, Nada Andelic^3^, Lasse Andreassen^4^, Audny Anke^5^, Anna Antoni^6^, Gérard Audibert^7^, Philippe Azouvi^8^, Maria Luisa Azzolini^9^, Ronald Bartels^10^, Pál Barzó^11^, Romuald Beauvais^12^, Ronny Beer^13^, Bo-Michael Bellander^14^, Antonio Belli^15^, Habib Benali^16^, Maurizio Berardino^17^, Luigi Beretta^9^, Morten Blaabjerg^18^, Peter Bragge^19^, Alexandra Brazinova^20^, Vibeke Brinck^21^, Joanne Brooker^22^, Camilla Brorsson^23^, Andras Buki^24^, Monika Bullinger^25^, Manuel Cabeleira^26^, Alessio Caccioppola^27^, Emiliana Calappi ^27^, Maria Rosa Calvi^9^, Peter Cameron^28^, Guillermo Carbayo Lozano^29^, Marco Carbonara^27^, Simona Cavallo^17^, Giorgio Chevallard^30^, Arturo Chieregato^30^, Giuseppe Citerio^31, 32^, Hans Clusmann^33^, Mark Coburn^34^, Jonathan Coles^35^, Jamie D. Cooper^36^, Marta Correia^37^, Amra Cović ^38^, Nicola Curry^39^, Endre Czeiter^24^, Marek Czosnyka^26^, Claire Dahyot-Fizelier^40^, Paul Dark^41^, Helen Dawes^42^, Véronique De Keyser^43^, Vincent Degos^16^, Francesco Della Corte^44^, Hugo den Boogert^10^, Bart Depreitere^45^, Ðula Ðilvesi^46^, Abhishek Dixit^47^, Emma Donoghue^22^, Jens Dreier^48^, Guy-Loup Dulière^49^, Ari Ercole^47^, Patrick Esser^42^, Erzsébet Ezer^50^, Martin Fabricius^51^, Valery L. Feigin^52^, Kelly Foks^53^, Shirin Frisvold^54^, Alex Furmanov^55^, Pablo Gagliardo^56^, Damien Galanaud^16^, Dashiell Gantner^28^, Guoyi Gao^57^, Pradeep George^58^, Alexandre Ghuysen^59^, Lelde Giga^60^, Ben Glocker^61^, Jagoš Golubovic^46^, Pedro A. Gomez ^62^, Johannes Gratz^63^, Benjamin Gravesteijn^64^, Francesca Grossi^44^, Russell L. Gruen^65^, Deepak Gupta^66^, Juanita A. Haagsma^64^, Iain Haitsma^67^, Raimund Helbok^13^, Eirik Helseth^68^, Lindsay Horton ^69^, Jilske Huijben^64^, Peter J. Hutchinson^70^, Bram Jacobs^71^, Stefan Jankowski^72^, Mike Jarrett^21^, Ji-yao Jiang^58^, Faye Johnson^73^, Kelly Jones^52^, Mladen Karan^46^, Angelos G. Kolias^70^, Erwin Kompanje^74^, Daniel Kondziella^51^, Evgenios Kornaropoulos^47^, Lars-Owe Koskinen^75^, Noémi Kovács^76^, Ana Kowark^77^, Alfonso Lagares^62^, Linda Lanyon^58^, Steven Laureys^78^, Fiona Lecky^79, 80^, Didier Ledoux^78^, Rolf Lefering^81^, Valerie Legrand^82^, Aurelie Lejeune^83^, Leon Levi^84^, Roger Lightfoot^85^, Hester Lingsma^64^, Andrew I.R. Maas^43^, Ana M. Castaño-León^62^, Marc Maegele^86^, Marek Majdan^20^, Alex Manara^87^, Geoffrey Manley^88^, Costanza Martino^89^, Hugues Maréchal^49^, Julia Mattern^90^, Catherine McMahon^91^, Béla Melegh^92^, David Menon^47^, Tomas Menovsky^43^, Ana Mikolic^64^, Benoit Misset^78^, Visakh Muraleedharan^58^, Lynnette Murray^28^, Ancuta Negru^93^, David Nelson^1^, Virginia Newcombe^47^, Daan Nieboer^64^, József Nyirádi^2^, Otesile Olubukola^79^, Matej Oresic^94^, Fabrizio Ortolano^27^, Aarno Palotie^95, 96, 97^, Paul M. Parizel^98^, Jean-François Payen^99^, Natascha Perera^12^, Vincent Perlbarg^16^, Paolo Persona^100^, Wilco Peul^101^, Anna Piippo-Karjalainen^102^, Matti Pirinen^95^, Dana Pisica^64^, Horia Ples^93^, Suzanne Polinder^64^, Inigo Pomposo^29^, Jussi P. Posti ^103^, Louis Puybasset^104^, Andreea Radoi ^105^, Arminas Ragauskas^106^, Rahul Raj^102^, Malinka Rambadagalla^107^, Isabel Retel Helmrich^64^, Jonathan Rhodes^108^, Sylvia Richardson^109^, Sophie Richter^47^, Samuli Ripatti^95^, Saulius Rocka^106^, Cecilie Roe^110^, Olav Roise^111, 112^, Jonathan Rosand^113^, Jeffrey V. Rosenfeld^114^, Christina Rosenlund^115^, Guy Rosenthal^55^, Rolf Rossaint^77^, Sandra Rossi^100^, Daniel Rueckert^61^, Martin Rusnák^116^, Juan Sahuquillo^105^, Oliver Sakowitz^90, 117^, Renan Sanchez-Porras^117^, Janos Sandor^118^, Nadine Schäfer^81^, Silke Schmidt^119^, Herbert Schoechl^120^, Guus Schoonman^121^, Rico Frederik Schou^122^, Elisabeth Schwendenwein^6^, Charlie Sewalt^64^, Ranjit D. Singh^101^, Toril Skandsen^123, 124^, Peter Smielewski^26^, Abayomi Sorinola^125^, Emmanuel Stamatakis^47^, Simon Stanworth^39^, Robert Stevens^126^, William Stewart^127^, Ewout W. Steyerberg^64^, ^128^, Nino Stocchetti^129^, Nina Sundström^130^, Riikka Takala^131^, Viktória Tamás^125^, Tomas Tamosuitis^132^, Mark Steven Taylor^20^, Braden Te Ao^52^, Olli Tenovuo^103^, Alice Theadom^52^, Matt Thomas^87^, Dick Tibboel^133^, Marjolein Timmers^74^, Christos Tolias^134^, Tony Trapani^28^, Cristina Maria Tudora^93^, Andreas Unterberg^90^, Peter Vajkoczy ^135^, Shirley Vallance^28^, Egils Valeinis^60^, Zoltán Vámos^50^, Mathieu van der Jagt^136^, Gregory Van der Steen^43^, Joukje van der Naalt^71^, Jeroen T.J.M. van Dijck ^101^, Inge A. van Erp^101^, Thomas A. van Essen^101^, Wim Van Hecke^137^, Caroline van Heugten^42^, Dominique Van Praag^138^, Ernest van Veen^64^, Thijs Vande Vyvere^137^, Roel P. J. van Wijk^101^, Alessia Vargiolu^32^, Emmanuel Vega^83^, Kimberley Velt^64^, Jan Verheyden^137^, Paul M. Vespa^139^, Anne Vik^123, 140^, Rimantas Vilcinis^132^, Victor Volovici^67^, Nicole von Steinbüchel^38^, Daphne Voormolen^64^, Petar Vulekovic^46^, Kevin K.W. Wang^141^, Daniel Whitehouse^47^, Eveline Wiegers^64^, Guy Williams^47^, Lindsay Wilson^69^, Stefan Winzeck^47^, Stefan Wolf^142^, Zhihui Yang^113^, Peter Ylén^143^, Alexander Younsi^90^, Frederick A. Zeiler^47, 144^, Veronika Zelinkova^20^, Agate Ziverte^60^, Tommaso Zoerle^27^

^1^Department of Physiology and Pharmacology, Section of Perioperative Medicine and Intensive Care, Karolinska Institutet, Stockholm, Sweden

^2^János Szentágothai Research Centre, University of Pécs, Pécs, Hungary

^3^Division of Surgery and Clinical Neuroscience, Department of Physical Medicine and Rehabilitation, Oslo University Hospital and University of Oslo, Oslo, Norway

^4^Department of Neurosurgery, University Hospital Northern Norway, Tromso, Norway

^5^Department of Physical Medicine and Rehabilitation, University Hospital Northern Norway, Tromso, Norway

^6^Trauma Surgery, Medical University Vienna, Vienna, Austria

^7^Department of Anesthesiology & Intensive Care, University Hospital Nancy, Nancy, France

^8^Raymond Poincare hospital, Assistance Publique – Hopitaux de Paris, Paris, France

^9^Department of Anesthesiology & Intensive Care, S Raffaele University Hospital, Milan, Italy

^10^Department of Neurosurgery, Radboud University Medical Center, Nijmegen, The Netherlands

^11^Department of Neurosurgery, University of Szeged, Szeged, Hungary

^12^International Projects Management, ARTTIC, Munchen, Germany

^13^Department of Neurology, Neurological Intensive Care Unit, Medical University of Innsbruck, Innsbruck, Austria

^14^Department of Neurosurgery & Anesthesia & intensive care medicine, Karolinska University Hospital, Stockholm, Sweden

^15^NIHR Surgical Reconstruction and Microbiology Research Centre, Birmingham, UK

^16^Anesthesie-Réanimation, Assistance Publique – Hopitaux de Paris, Paris, France

^17^Department of Anesthesia & ICU, AOU Città della Salute e della Scienza di Torino - Orthopedic and Trauma Center, Torino, Italy

^18^Department of Neurology, Odense University Hospital, Odense, Denmark

^19^BehaviourWorks Australia, Monash Sustainability Institute, Monash University, Victoria, Australia

^20^Department of Public Health, Faculty of Health Sciences and Social Work, Trnava University, Trnava, Slovakia

^21^Quesgen Systems Inc., Burlingame, CA, United States

^22^Australian & New Zealand Intensive Care Research Centre, Department of Epidemiology and Preventive Medicine, School of Public Health and Preventive Medicine, Monash University, Melbourne, Australia

^23^Department of Surgery and Perioperative Science, Umeå University, Umeå, Sweden

^24^Department of Neurosurgery, Medical School, University of Pécs, Hungary and Neurotrauma Research Group, János Szentágothai Research Centre, University of Pécs, Hungary

^25^Department of Medical Psychology, Universitätsklinikum Hamburg-Eppendorf, Hamburg, Germany

^26^Brain Physics Lab, Division of Neurosurgery, Dept of Clinical Neurosciences, University of Cambridge, Addenbrooke's Hospital, Cambridge, United Kingdom

^27^Neuro ICU, Fondazione IRCCS Cà Granda Ospedale Maggiore Policlinico, Milan, Italy

^28^ANZIC Research Centre, Monash University, Department of Epidemiology and Preventive Medicine, Melbourne, Victoria, Australia

^29^Department of Neurosurgery, Hospital of Cruces, Bilbao, Spain

^30^NeuroIntensive Care, Niguarda Hospital, Milan, Italy

^31^School of Medicine and Surgery, Università Milano Bicocca, Milano, Italy

^32^NeuroIntensive Care, ASST di Monza, Monza, Italy

^33^Department of Neurosurgery, Medical Faculty RWTH Aachen University, Aachen, Germany

^34^Department of Anesthesiology and Intensive Care Medicine, University Hospital Bonn, Bonn, Germany

^35^Department of Anesthesia & Neurointensive Care, Cambridge University Hospital NHS Foundation Trust, Cambridge, United Kingdom

^36^School of Public Health & PM, Monash University and The Alfred Hospital, Melbourne, VIC, Australia

^37^Radiology/MRI department, MRC Cognition and Brain Sciences Unit, Cambridge, United Kingdom

^38^Institute of Medical Psychology and Medical Sociology, Universitätsmedizin Göttingen, Göttingen, Germany

^39^Oxford University Hospitals NHS Trust, Oxford, United Kingdom

^40^Intensive Care Unit, CHU Poitiers, Potiers, France

^41^University of Manchester NIHR Biomedical Research Centre, Critical Care Directorate, Salford Royal Hospital NHS Foundation Trust, Salford, United Kingdom

^42^Movement Science Group, Faculty of Health and Life Sciences, Oxford Brookes University, Oxford, United Kingdom

^43^Department of Neurosurgery, Antwerp University Hospital and University of Antwerp, Edegem, Belgium

^44^Department of Anesthesia & Intensive Care, Maggiore Della Carità Hospital, Novara, Italy

^45^Department of Neurosurgery, University Hospitals Leuven, Leuven, Belgium

^46^Department of Neurosurgery, Clinical centre of Vojvodina, Faculty of Medicine, University of Novi Sad, Novi Sad, Serbia

^47^Division of Anaesthesia, University of Cambridge, Addenbrooke's Hospital, Cambridge, United Kingdom

^48^Center for Stroke Research Berlin, Charité – Universitätsmedizin Berlin, corporate member of Freie Universität Berlin, Humboldt-Universität zu Berlin, and Berlin Institute of Health, Berlin, Germany

^49^Intensive Care Unit, CHR Citadelle, Liège, Belgium

^50^Department of Anaesthesiology and Intensive Therapy, University of Pécs, Pécs, Hungary

^51^Departments of Neurology, Clinical Neurophysiology and Neuroanesthesiology, Region Hovedstaden Rigshospitalet, Copenhagen, Denmark

^52^National Institute for Stroke and Applied Neurosciences, Faculty of Health and Environmental Studies, Auckland University of Technology, Auckland, New Zealand

^53^Department of Neurology, Erasmus MC, Rotterdam, the Netherlands

^54^Department of Anesthesiology and Intensive care, University Hospital Northern Norway, Tromso, Norway

^55^Department of Neurosurgery, Hadassah-hebrew University Medical center, Jerusalem, Israel

^56^Fundación Instituto Valenciano de Neurorrehabilitación (FIVAN), Valencia, Spain

^57^Department of Neurosurgery, Shanghai Renji hospital, Shanghai Jiaotong University/school of medicine, Shanghai, China

^58^Karolinska Institutet, INCF International Neuroinformatics Coordinating Facility, Stockholm, Sweden

^59^Emergency Department, CHU, Liège, Belgium

^60^Neurosurgery clinic, Pauls Stradins Clinical University Hospital, Riga, Latvia

^61^Department of Computing, Imperial College London, London, United Kingdom

^62^Department of Neurosurgery, Hospital Universitario 12 de Octubre, Madrid, Spain

^63^Department of Anesthesia, Critical Care and Pain Medicine, Medical University of Vienna, Austria

^64^Department of Public Health, Erasmus Medical Center-University Medical Center, Rotterdam, The Netherlands

^65^College of Health and Medicine, Australian National University, Canberra, ACT, Australia

^66^Department of Neurosurgery, Neurosciences Centre & JPN Apex trauma centre, All India Institute of Medical Sciences, New Delhi, India

^67^Department of Neurosurgery, Erasmus MC, Rotterdam, the Netherlands

^68^Department of Neurosurgery, Oslo University Hospital, Oslo, Norway

^69^Division of Psychology, University of Stirling, Stirling, United Kingdom

^70^Division of Neurosurgery, Department of Clinical Neurosciences, Addenbrooke's Hospital & University of Cambridge, Cambridge, United Kingdom

^71^Department of Neurology, University of Groningen, University Medical Center Groningen, Groningen, Netherlands

^72^Neurointensive Care, Sheffield Teaching Hospitals NHS Foundation Trust, Sheffield, United Kingdom

^73^Salford Royal Hospital NHS Foundation Trust Acute Research Delivery Team, Salford, United Kingdom

^74^Department of Intensive Care and Department of Ethics and Philosophy of Medicine, Erasmus Medical Center, Rotterdam, The Netherlands

^75^Department of Clinical Neuroscience, Neurosurgery, Umeå University, Umeå, Sweden

^76^Hungarian Brain Research Program - Grant No. KTIA_13_NAP-A-II/8, University of Pécs, Pécs, Hungary

^77^Department of Anaesthesiology, University Hospital of Aachen, Aachen, Germany

^78^Cyclotron Research Center, University of Liège, Liège, Belgium

^79^Centre for Urgent and Emergency Care Research (CURE), Health Services Research Section, School of Health and Related Research (ScHARR), University of Sheffield, Sheffield, UK

^80^Emergency Department, Salford Royal Hospital, Salford, United Kingdom

^81^Institute of Research in Operative Medicine (IFOM), Witten/Herdecke University, Cologne, Germany

^82^VP Global Project Management CNS, ICON, Paris, France

^83^Department of Anesthesiology-Intensive Care, Lille University Hospital, Lille, France

^84^Department of Neurosurgery, Rambam Medical Center, Haifa, Israel

^85^Department of Anesthesiology & Intensive Care, University Hospitals Southhampton NHS Trust, Southhampton, United Kingdom

^86^Cologne-Merheim Medical Center (CMMC), Department of Traumatology, Orthopedic Surgery and Sportmedicine, Witten/Herdecke University, Cologne, Germany

^87^Intensive Care Unit, Southmead Hospital, Bristol, Bristol, Uinted Kingdom

^88^Department of Neurological Surgery, University of California, San Francisco, CA, United States

^89^Department of Anesthesia & Intensive Care, M. Bufalini Hospital, Cesena, Italy

^90^Department of Neurosurgery, University Hospital Heidelberg, Heidelberg, Germany

^91^Department of Neurosurgery, The Walton centre NHS Foundation Trust, Liverpool, United Kingdom

^92^Department of Medical Genetics, University of Pécs, Pécs, Hungary

^93^Department of Neurosurgery, Emergency County Hospital Timisoara, Timisoara, Romania

^94^School of Medical Sciences, Örebro University, Örebro, Sweden

^95^Institute for Molecular Medicine Finland, University of Helsinki, Helsinki, Finland

^96^Analytic and Translational Genetics Unit, Department of Medicine; Psychiatric & Neurodevelopmental Genetics Unit, Department of Psychiatry; Department of Neurology, Massachusetts General Hospital, Boston, MA, United States

^97^Program in Medical and Population Genetics; The Stanley Center for Psychiatric Research, The Broad Institute of MIT and Harvard, Cambridge, MA, United States

^98^Department of Radiology, University of Antwerp, Edegem, Belgium

^99^Department of Anesthesiology & Intensive Care, University Hospital of Grenoble, Grenoble, France

^100^Department of Anesthesia & Intensive Care, Azienda Ospedaliera Università di Padova, Padova, Italy

^101^Dept. of Neurosurgery, Leiden University Medical Center, Leiden, The Netherlands and Dept. of Neurosurgery, Medical Center Haaglanden, The Hague, Netherlands

^102^Department of Neurosurgery, Helsinki University Central Hospital

^103^Division of Clinical Neurosciences, Department of Neurosurgery and Turku Brain Injury Centre, Turku University Hospital and University of Turku, Turku, Finland

^104^Department of Anesthesiology and Critical Care, Pitié -Salpêtrière Teaching Hospital, Assistance Publique, Hôpitaux de Paris and University Pierre et Marie Curie, Paris, France

^105^Neurotraumatology and Neurosurgery Research Unit (UNINN), Vall d'Hebron Research Institute, Barcelona, Spain

^106^Department of Neurosurgery, Kaunas University of technology and Vilnius University, Vilnius, Lithuania

^107^Department of Neurosurgery, Rezekne Hospital, Latvia

^108^Department of Anaesthesia, Critical Care & Pain Medicine NHS Lothian & University of Edinburg, Edinburgh, United Kingdom

^109^Director, MRC Biostatistics Unit, Cambridge Institute of Public Health, Cambridge, United Kingdom

^110^Department of Physical Medicine and Rehabilitation, Oslo University Hospital/University of Oslo, Oslo, Norway

^111^Division of Orthopedics, Oslo University Hospital, Oslo, Norway

^112^Institue of Clinical Medicine, Faculty of Medicine, University of Oslo, Oslo, Norway

^113^Broad Institute, Cambridge MA Harvard Medical School, Boston MA, Massachusetts General Hospital, Boston MA, United States

^114^National Trauma Research Institute, The Alfred Hospital, Monash University, Melbourne, VIC, Australia

^115^Department of Neurosurgery, Odense University Hospital, Odense, Denmark

^116^International Neurotrauma Research Organisation, Vienna, Austria

^117^Klinik für Neurochirurgie, Klinikum Ludwigsburg, Ludwigsburg, Germany

^118^Division of Biostatistics and Epidemiology, Department of Preventive Medicine, University of Debrecen, Debrecen, Hungary

^119^Department Health and Prevention, University Greifswald, Greifswald, Germany

^120^Department of Anaesthesiology and Intensive Care, AUVA Trauma Hospital, Salzburg, Austria

^121^Department of Neurology, Elisabeth-TweeSteden Ziekenhuis, Tilburg, Netherlands

^122^Department of Neuroanesthesia and Neurointensive Care, Odense University Hospital, Odense, Denmark

^123^Department of Neuromedicine and Movement Science, Norwegian University of Science and Technology, NTNU, Trondheim, Norway

^124^Department of Physical Medicine and Rehabilitation, St. Olavs Hospital, Trondheim University Hospital, Trondheim, Norway

^125^Department of Neurosurgery, University of Pécs, Pécs, Hungary

^126^Division of Neuroscience Critical Care, John Hopkins University School of Medicine, Baltimore, USA

^127^Department of Neuropathology, Queen Elizabeth University Hospital and University of Glasgow, Glasgow, United Kingdom

^128^Dept. of Department of Biomedical Data Sciences, Leiden University Medical Center, Leiden, Netherlands

^129^Department of Pathophysiology and Transplantation, Milan University, and Neuroscience ICU, Fondazione IRCCS Cà Granda Ospedale Maggiore Policlinico, Milano, Italy

^130^Department of Radiation Sciences, Biomedical Engineering, Umeå University, Umeå, Sweden

^131^Perioperative Services, Intensive Care Medicine and Pain Management, Turku University Hospital and University of Turku, Turku, Finland

^132^Department of Neurosurgery, Kaunas University of Health Sciences, Kaunas, Lithuania

^133^Intensive Care and Department of Pediatric Surgery, Erasmus Medical Center, Sophia Children's Hospital, Rotterdam, The Netherlands

^134^Department of Neurosurgery, Kings college London, London, United Kingdom

^135^Neurologie, Neurochirurgie und Psychiatrie, Charité – Universitätsmedizin Berlin, Berlin, Germany

^136^Department of Intensive Care Adults, Erasmus MC– University Medical Center Rotterdam, Rotterdam, the Netherlands

^137^icoMetrix NV, Leuven, Belgium

^138^Psychology Department, Antwerp University Hospital, Edegem, Belgium

^139^Director of Neurocritical Care, University of California, Los Angeles, United States

^140^Department of Neurosurgery, St.Olavs Hospital, Trondheim University Hospital, Trondheim, Norway

^141^Department of Emergency Medicine, University of Florida, Gainesville, Florida, United States

^142^Department of Neurosurgery, Charité – Universitätsmedizin Berlin, corporate member of Freie Universität Berlin, Humboldt-Universität zu Berlin, and Berlin Institute of Health, Berlin, Germany

^143^VTT Technical Research Centre, Tampere, Finland

^144^Section of Neurosurgery, Department of Surgery, Rady Faculty of Health Sciences, University of Manitoba, Winnipeg, MB, Canada

## Conflict of interest

The authors declare that the research was conducted in the absence of any commercial or financial relationships that could be construed as a potential conflict of interest.

## Publisher's note

All claims expressed in this article are solely those of the authors and do not necessarily represent those of their affiliated organizations, or those of the publisher, the editors and the reviewers. Any product that may be evaluated in this article, or claim that may be made by its manufacturer, is not guaranteed or endorsed by the publisher.
