# Supplementary material for: Concomitant spine trauma in patients with traumatic brain injury: Patient characteristics and outcomes
Source: Front Neurol. 2022 Aug 18;13:861688. doi: 10.3389/fneur.2022.861688 (PMC9436444; doi:10.3389/fneur.2022.861688)
Supplement: Supplementary file 1 [file Table_1.docx]

**Supplement Table 1:** The data visualized in Figure 2 is presented in further detail (with more variables included).

| Variable | Value | Missing data (n) |
| --- | --- | --- |
| Age | 53 (37-66) | 0 |
| Female sex | 48 (29%) | 0 |
| Injury cause  Road-traffic incident  Incidental falls  Other non-intentional inj.  Violence/assault  Other | 68 (43%)  77 (49%)  6 (4%)  3 (2%)  4 (3%) | 6 |
| Alcohol intoxication  None  Confirmed  Suspected | 106 (65%)  26 (19%)  13 (9%) | 19 |
| Arrival method  Ambulance  Helicopter  Medical mobile team  Walk-in/Drop-off  Other | 123 (76%)  19 (12%)  10 (6%)  9 (6%)  1 (<%) | 2 |
| Prehospital intubation | 33 (22%) | 23 |
| Referral  Primary study center  Secondary referral | 141 (86%)  23 (14%) | 0 |
| GCS  13-15  9-12  3-8 | 101 (62%)  16 (10%)  42 (26%) | 5 |
| Spine injury (AIS >= 3)  Cervical  Thoracic  Lumbar  Multiple | 104 (63%)  51 (31%)  32 (20%)  21 (13%) | 0 |
| Clinical care pathway  Discharge  Admission  ICU admission | 8 (5%)  63 (38%)  93 (57%) | 0 |
| ICU Reason  Mechanical ventilation  Neurological observation  Neurological operations  4  6  3  Other | 40 (43%)  22 (24%)  13 (14%)  7 (8%)  3 (3%)  1 (1%)  7 (8%) | 0 |
| Complications  Respiratory  Seizures  Cardiac  UTI | 14 (12%)  5 (4%)  4 (3%)  6 (5%) | 46 |
| Length of stay (days) | 9 (3-20) | 1 |
| Discharge aim  Home  Rehabilitation facility  Other hospital | 70 (56%)  26 (21%)  24 (19%) | 39 |
| Outcome at six months  Fatal  Unfavourable (GOSE <5)  Good (GOSE 7-8) | 18 (11%)  48 (29%)  59 (42%) | 25 |

AIS: Abbreviated injury scale; GCS: Glasgow Outcome Scale; GOSe: Glasgow Outcome Scale Extended; ICU: Intensive care unit; UTI: Urinary tract infection.
