# Supplementary material for: Concomitant spine trauma in patients with traumatic brain injury: Patient characteristics and outcomes
Source: Front Neurol. 2022 Aug 18;13:861688. doi: 10.3389/fneur.2022.861688 (PMC9436444; doi:10.3389/fneur.2022.861688)
Supplement: Supplementary file 2 [file Table_2.docx]

**Supplement Table 2:** Balance statistics in the TBI with isolated ST cohort after matching across all imputed datasets.

| Variable | Mean adj. difference | | | |
| --- | --- | --- | --- | --- |
|  | TBI + isolated ST | TBI + ST + syst. injuries | mTBI + isolated ST | mTBI + ST + syst. injuries |
| Age | -0.0477 | -0.0122 | -0.0505 | 0.0146 |
| Sex | -0.0172 | -0.0213 | -0.0246 | -0.0715 |
| GCS  3  4  5  6  7  8  9  10  11  12  13  14  15 | 0.0155  -0.0007  -0.0073  0.0047  -0.0065  -0.0005  -0.0008  -0.0013  -0.0004  0.0050  0.0138  -0.0002  -0.0213 | -0.0033  0.0053  0.0029  -0.0044  0.0029  -0.0019  0.0037  0.0027  -0.0011  -0.0055  0.0011  0.0072  -0.0096 | 0.0250  0.0169  -0.0419 | 0.0142  0.0150  -0.0292 |
| Cranial surgery  No  Yes | -0.0151  0.0151 | 0.0086  -0.0086 | 0.0123 | 0.0113 |
| CT abnormality  No  Yes  Uninterpretable | -0.0095  0.0098  -0.0003 | -0.0014  -0.0061  0.0075 | -0.0190  0.0064  0.0126 | -0.0140  -0.0036  0.0175 |
| ASA class  1  2  3  4  Unknown | 0.0149  -0.0244  0.0046  0.0020  0.0029 | -0.0203  0.0036  0.0148  0.0014  0.0005 | 0.0111  -0.0298  0.0086  0.0050  0.0051 | -0.0164  -0.0057  0.0191  0.0046  -0.0015 |

The difference in proportion and standardized mean difference (averaged across all imputed datasets) is displayed for binary and continuous variables, respectively.

ASA = American Society of Anesthesiologists; CT = computed tomography; GCS = Glasgow Coma scale.
