# Supplementary material for: Concomitant spine trauma in patients with traumatic brain injury: Patient characteristics and outcomes
Source: Front Neurol. 2022 Aug 18;13:861688. doi: 10.3389/fneur.2022.861688 (PMC9436444; doi:10.3389/fneur.2022.861688)
Supplement: Supplementary file 3 [file Table_3.docx]

**Supplement Table 3:** Comparison of variables in patients with TBI + isolated ST (A), TBI + ST + systemic injuries (B), mTBI + isolated ST (C), and mTBI + ST + systemic injuries (d) vs. the respective propensity-score-matched patients. As matching was performed in all 100 imputed datasets, one of those datasets from each matched cohort was respectively chosen by a random generator and displayed in the tables A-D for demonstration.

**A) TBI + isolated ST (dataset 34 of the 100 imputed datasets)**

| Variable | TBI + isolated ST | TBI without ST | p-value |
| --- | --- | --- | --- |
| Age | 53 (37-66) | 53 (36-69) | 0.678 |
| Female sex | 48 (29%) | 43 (26%) | 0.622 |
| GCS  13-15  9-12  3-8 | 102 (62%)  18 (11%)  44 (27%) | 99 (60%)  18 (11%)  47 (29%) | 0.902 |
| Cranial surgery | 38 (23%) | 35 (21%) | 0.791 |
| CT abnormality  Yes  Uninterpretable | 106 (65%)  7 (4%) | 101 (62%)  9 (5%) | 0.876 |
| ASA class  1  2  3  4  Unknown | 81 (49%)  63 (38%)  14 (9%)  1 (<1%)  5 (3%) | 75 (46%)  67 (41%)  17 (10%)  0 (0%)  5 (3%) | 0.889 |

**B) TBI + ST + systemic injuries (dataset 78 of the 100 imputed datasets)**

| Variable | TBI + ST | TBI without ST | p-value |
| --- | --- | --- | --- |
| Age | 50 (31-61) | 49 (32-64) | 0.705 |
| Female sex | 70 (23%) | 72 (24%) | 0.923 |
| GCS  13-15  9-12  3-8 | 119 (40%)  34 (11%)  147 (49%) | 117 (39%)  35 (12%)  148 (49%) | 0.983 |
| Cranial surgery | 73 (24%) | 69 (23%) | 0.773 |
| CT abnormality  Yes  Uninterpretable | 225 (75%)  11 (4%) | 229 (76%)  7 (2%) | 0.630 |
| ASA class  1  2  3  4  Unknown | 170 (57%)  96. (32%)  19 (6%)  2 (<1%)  13 (4%) | 177 (59%)  95 (32%)  13 (4%)  2 (<1%)  13 (4%) | 0.868 |

**C) mTBI + isolated ST (dataset 95 of the 100 imputed datasets)**

| Variable | TBI + ST | TBI without ST | p-value |
| --- | --- | --- | --- |
| Age | 54 (39–68) | 55 (42–67) | 0.728 |
| Female sex | 34 (33%) | 31 (31%) | 0.763 |
| GCS  13-15  9-12  3-8 | 101 (100%) | 101 (100%) | - |
| Cranial surgery  Yes | 7 (7%) | 4 (4%) | 0.535 |
| CT abnormality  Yes  Uninterpretable | 43 (43%)  7 (7%) | 45 (45%)  5 (5%) | 0.827 |
| ASA class  1  2  3  4  Unknown | 50 (50%)  41 (41%)  7 (7%)  1 (<1%)  2 (2%) | 48 (48%)  42 (42%)  8 (8%)  1 (<1%)  2 (2%) | 0.991 |

**D) mTBI + ST + systemic injuries (dataset 68 of the 100 imputed datasets)**

| Variable | TBI + ST | TBI without ST | p-value |
| --- | --- | --- | --- |
| Age | 52 (35–62) | 52 (37–64) | 0.691 |
| Female sex | 36 (31%) | 25 (21%) | 0.137 |
| GCS  13-15  9-12  3-8 | 118 (100%) | 118 (100%) | - |
| Cranial surgery | 10 (8%) | 8 (7%) | 0.806 |
| CT abnormality  Yes  Uninterpretable | 66 (56%)  4 (3%) | 66 (56%)  5 (4%) | 1.000 |
| ASA class  1  2  3  4  Unknown | 66 (56%)  41 (35%)  7 (6%)  1 (<1%)  3 (3%) | 73 (62%)  36 (31%)  6 (5%)  1 (<1%)  2 (2%) | 0.906 |

ASA = American Society of Anesthesiologists; CT = computed tomography; GCS = Glasgow Coma scale; ST = spine trauma.
