# Supplementary material for: Concomitant spine trauma in patients with traumatic brain injury: Patient characteristics and outcomes
Source: Front Neurol. 2022 Aug 18;13:861688. doi: 10.3389/fneur.2022.861688 (PMC9436444; doi:10.3389/fneur.2022.861688)
Supplement: Supplementary file 4 [file Table_4.docx]

**Supplement Table 4:** Logistic regression analysis of full recovery, unfavorable outcome, and mortality in patients with (m)TBI patients and concomitant ST (isolated or non-isolated).

**A) Entire cohort (all TBI severities)**

| Predictor | Outcome | Beta coefficient | Standard error | p-value |
| --- | --- | --- | --- | --- |
| Isolated spine trauma* | Full recovery | -0.371 | 0.215 | 0.084 |
|  | Unfavorable outcome | 0.305 | 0.223 | 0.184 |
|  | Mortality | -0.292 | 0.316 | 0.355 |
| Spine trauma plus systemic injury* | Full recovery | -1.037 | 0.172 | <0.001 |
|  | Unfavorable outcome | 0.493 | 0.166 | 0.003 |
|  | Mortality | -0.195 | 0.211 | 0.355 |

**B) Only mTBI**

| Predictor | Outcome | Beta coefficient | Standard error | p-value |
| --- | --- | --- | --- | --- |
| Isolated spine trauma* | Full recovery | -0.507 | 0.248 | 0.042 |
|  | Unfavorable outcome | 0.770 | 0.318 | 0.016 |
| Spine trauma plus systemic injury* | Full recovery | -1.345 | 0.218 | <0.001 |
|  | Unfavorable outcome | 1.150 | 0.276 | <0.001 |

* All models were adjusted for age, sex, cranial surgery, CT abnormality, baseline GCS, and ASA class.
